# Effects of Salinity on the Growth and Nutrition of Taro (*Colocasia esculenta*): Implications for Food Security

**DOI:** 10.3390/plants10112319

**Published:** 2021-10-28

**Authors:** Georgia R. Lloyd, Akane Uesugi, Roslyn M. Gleadow

**Affiliations:** 1 School of Biological Sciences, Monash University, Clayton, Melbourne, VIC 3800, Australia; georgia.lloyd@monash.edu (G.R.L.); akane.uesugi@rmit.edu.au (A.U.); 2 School of Biosciences and Food Technology, RMIT, Bundoora Campus, 264 Plenty Road, Mill Park, VIC 3082, Australia

**Keywords:** calcium oxalate, sea level, secondary metabolites, plant defence, tuberous crops, Pacific Islands, salinity

## Abstract

Taro (*Colocasia esculenta* (L.) Schott) is a staple food crop in the Asia-Pacific region in areas where rising sea levels are threatening agricultural production. However, little is known about its response to salinity. In this study, we investigated the effects of salinity on the growth, morphology, physiology, and chemical traits of taro to predict the impacts of rising sea levels on taro production and nutritional value in the Pacific. We grew taro (approximately 4 months old) with a range of NaCl treatments (0–200 mM) for 12 weeks. Full nutrient, micronutrient, and secondary metabolite analyses were conducted, including measures of calcium oxalate (CaOx), an irritant that reduces palatability. Significant reductions in growth and biomass were observed at and above 100 mM NaCl. Concentrations of macro- and micronutrients, including sodium, were higher on a per mass basis in corms of plants experiencing salt stress. Foliar sodium concentrations remained stable, indicating that taro may utilize a salt exclusion mechanism. There was a large amount of individual variation in the concentrations of oxalate and phenolics, but overall, the concentrations were similar in the plants grown with different levels of salt. The total contents of CaOx and phenolics decreased in plants experiencing salt stress. Taro’s ability to survive and produce corms when watered with a 200 mM NaCl solution places it among the salt-tolerant non-halophytes. The nutritional quality of the crop is only marginally affected by salt stress. Taro is, therefore, likely to remain a useful staple in the Pacific region in the future.

## 1. Introduction

Rising sea levels pose a major challenge for the food security and livelihood of communities around the world, particularly in low-lying regions, such as the Pacific [1,2]. Salinity can have deleterious effects on plant growth and productivity, and nutritional quality, and as such, is a major environmental constraint to crop production [1,3,4]. With sea levels in the Pacific rising by as much as 10 mm each year [2], it is important to assess the tolerance of major crop plants in the region to salinity in order to prepare for the future.

Root and tuber crops are important staples throughout the world. This is particularly true in the Pacific Islands, where, aside from bananas and breadfruit, the main staples are yams, taro, cassava, and sweet potato [5]. Root and tuber crops are thought to be predominately salt-sensitive, with significant reductions in growth and yield under moderate salinity [1,6,7]. The starchy underground corms of taro (*Colocasia esculenta* (L.) Schott) are a staple food for over 200 million people in the Indo-Pacific region and are the main source of carbohydrates in the Pacific Islands [8,9,10,11]. Taro is also consumed widely in Africa [12]. However, despite its regional importance, taro is a neglected crop, and little is known about taro physiology or how it responds to environmental stresses of any kind [11,13].

Plants respond to salinity (NaCl) in multiple ways. Some minimize the deleterious effects through ion exclusion, which prevents Na^+^ and Cl^−^ transport to, and accumulation in, above-ground tissues, particularly the leaves. Other plants exhibit tissue tolerance, which prevents cell damage by the compartmentalization of Na^+^ and Cl^−^ in vacuoles [14,15,16,17]. The sodium concentration in plant tissues can be used to evaluate the mechanisms of tolerance; plants grown with high salinity and that have low concentrations of NaCl in their tissues may possess mechanisms to exclude NaCl and are classed as ‘excluders’ [3,18]. Conversely, if plants grown with high salinity are found to have high concentrations of NaCl in healthy organs, such as the leaves, that would indicate that there is tolerance at the tissue level, referred to as ‘tissue tolerance’ [16].

Salinity is known to affect plant nutritional quality and palatability by altering the levels of secondary metabolites, protein, and micronutrients [19]. Salt stress has been found to increase some secondary metabolites in non-tuberous crops [19,20]. However, little is known about the effects of salinity on tuberous crops. Gleadow, Pegg, and Blomstedt [1] found that there was generally an increase in the concentrations of cyanogenic glucosides in the tuberous roots of cassava in response to salinity, although the amount depended on the age of the plant and the degree of stress. Taro contains several chemical compounds that reduce its palatability and increase its toxicity. Taro is one of the aroid lilies (*Araceae*), a group notable for storing CaOx crystals. CaOx crystals make the leaves and corms highly acrid and can cause irritation and damage to the mouth, throat, and gut unless thoroughly washed and cooked before consumption [9,11,21]. Some taro cultivars also contain the cyanogenic glycoside triglochinin, a nitrogen-based phytoanticipin that breaks down to release toxic hydrogen cyanide on tissue disruption [22,23]. To ensure taro will continue to be suitable for human consumption in areas affected by rising sea levels, it is important to investigate the impact of salinity on these anti-nutritional factors, particularly calcium oxalate production, for which the effect of stress is completely unknown. Only a handful of studies have examined the salinity tolerance of taro, giving widely divergent results in terms of growth, and none of them measured the effect on CaOx [13,24].

We tested the impact of salinity on the growth and nutritional value of taro plants by watering with five different concentrations of NaCl (0, 50, 100, 150, and 200 mM) for 12 weeks. We found that taro was relatively salt-tolerant in that while high salt concentrations negatively affected growth and plant biomass, plants survived salinities up to 200 mM, with minimal effects on palatability. We conclude that taro is likely to continue to be a suitable food security option in the Pacific region in the future.

## 2. Results

### 2.1. Plant Growth and Biomass

**Figure 1:**
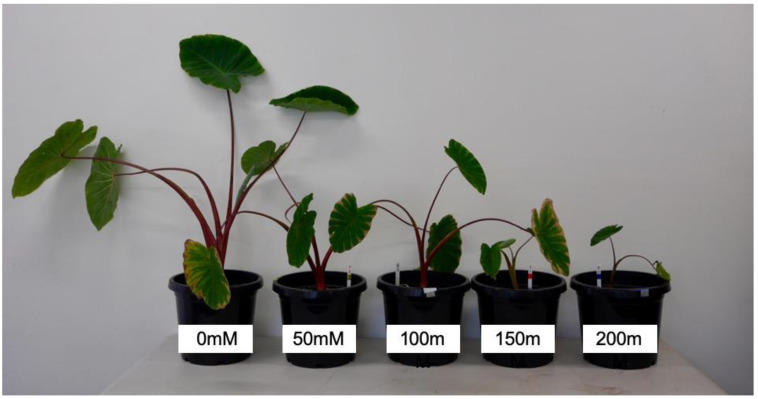
Representative 30-week-old taro plants at the time of harvest after being supplied with five different concentrations of salt (0, 50, 100, 150, and 200 mM NaCl) for 12 weeks.

**Figure 2:**
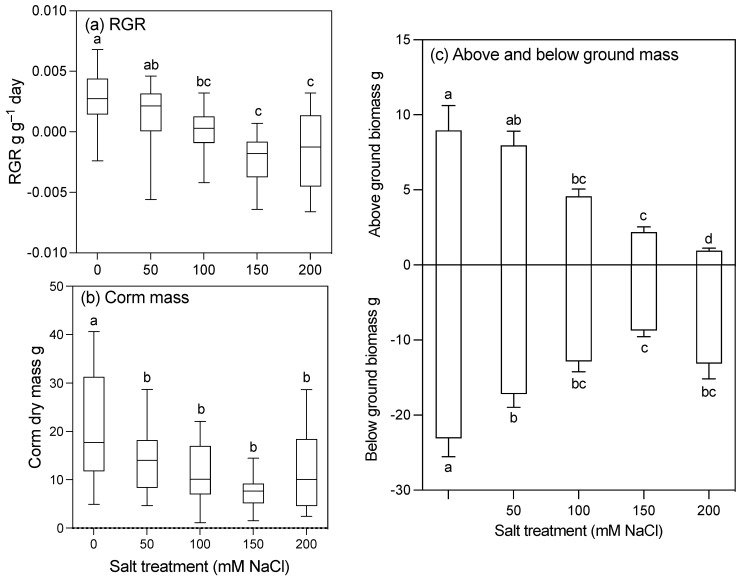
(**a**) Relative growth rate, (**b**) corm mass (g dry weight), and (**c**) total above- and below-ground biomasses (g dry weight) of 30-week-old taro plants grown for 12 weeks with five different concentrations of sodium chloride. Box plots show the medians of 18 replicates, range, and upper and lower quartiles. Bars are the means of 18 replicates ± 1 SE. Boxes and columns for the same parameter with the same letter are not significantly different at *p* < 0.05.

**Figure 3:**
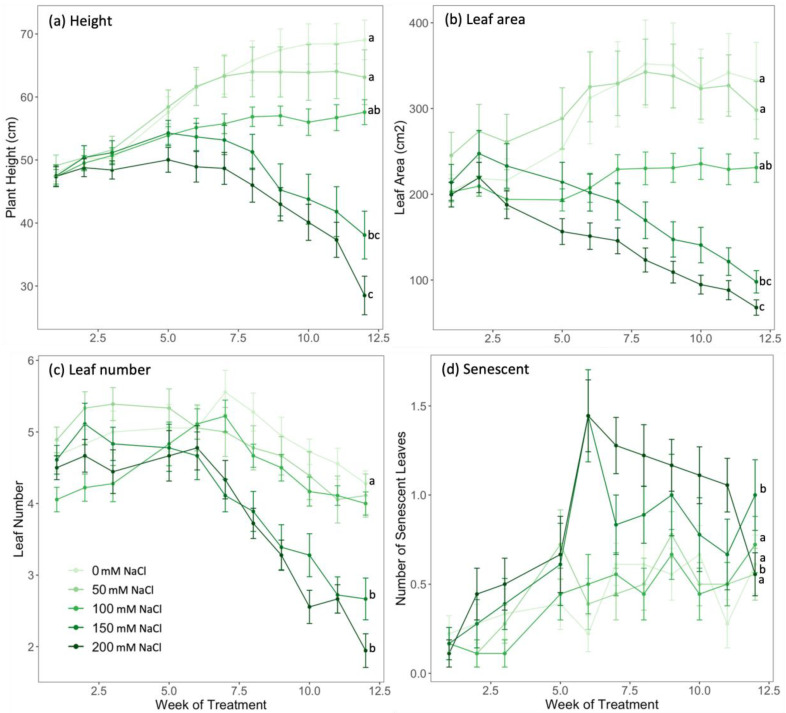
(**a**) Height, (**b**) leaf area, (**c**) leaf number, and (**d**) number of senescent leaves of taro plants supplied with five different concentrations of salt (0, 50, 100, 150, and 200 mM NaCl) for 12 weeks. Values are the means of 18 replicates ± 1 SE. The same plants were measured each week. Points for the same parameter with the same letter are not significantly different at *p* < 0.05. Pairwise differences were significant for weeks 4–12 of treatment.

Salt significantly reduced the growth rate of taro, but plants were not killed (Figure 1). Overall, plants were smaller (height, biomass) and had lower RGRs with increasing concentrations of NaCl (Table 1, Figure 2 and Figure 3a). However, even plants subjected to 200 mM NaCl were still alive at the end of the 12-week treatment. While total plant biomass was lower (25–66%) in all salt treatments (50, 100, 150, and 200 mM NaCl) compared to the control, the impact on plant growth parameters was minimal at 50 mM (Figure 2c). This reduction in biomass was the result of significant declines in both above- and below-ground biomass across all plant parts (Table 1, Figure 2c). Corm mass was significantly reduced across salt treatments, with a 38–43% reduction in the mean corm mass in plants in the 200 mM NaCl treatment group compared to the control (Table 1, Figure 2b). Despite this, the root/shoot ratio of plants in the 200 mM NaCl treatment group was higher than in the control plants (Table 1), primarily due to the decrease in the above-ground biomass.

There were significant interactions between the salt treatments and the length of the treatments for height, leaf area, and number (*p* ≤ 0.001), but no clear effect of salt was shown until week 3 (Figure 3). The leaf area was lower in plants in the 150 and 200 mM NaCl treatment groups compared to the other salt treatments (Figure 3b). These plants also had more senescent leaves over the course of the experiment than those in other treatment groups (Figure 3d). There was no significant difference in the leaf area ratio (LAR) between treatments, but the net assimilation rate (NAR) was significantly higher in plants in the 200 mM NaCl treatment group (Table 1).

Plant suckers (side shoots) were analysed separately from the main plants to determine whether their formation or biomass was influenced by the salt treatments. The number of suckers per plant, the total sucker biomass, the percentage of the total biomass in suckers, and the ratio of sucker corm mass-to-main corm mass did not differ significantly between the salt treatments (Supplementary Table S1).

### 2.2. Photosynthetic Parameters

**Figure 4:**
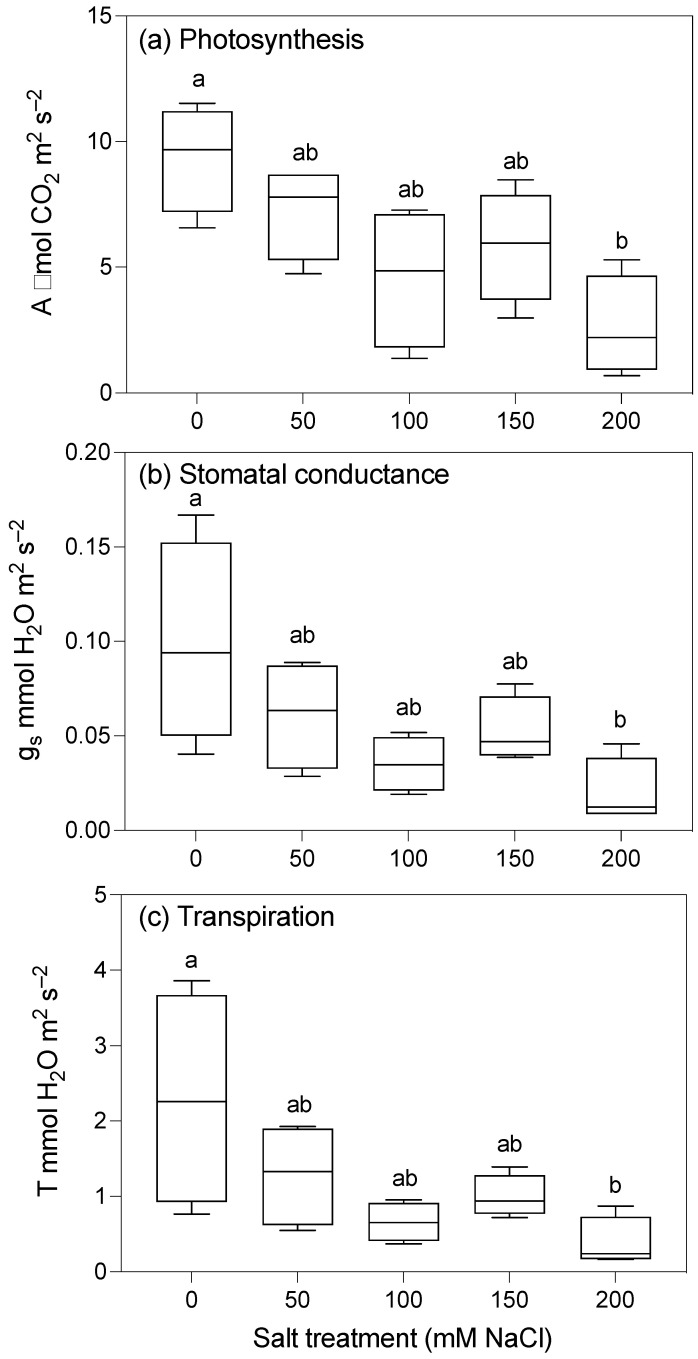
Gas exchange parameters of taro plants grown for 12 weeks with five different concentrations of salt. (**a**) A, Assimilation; (**b**) gs, stomatal conductance; (**c**) T, transpiration. Figures show the medians of four replicates, range, and upper and lower quartiles. Boxes for the same parameter with the same letter are not significantly different at *p* < 0.05.

The photosynthetic rate (i.e., the carbon assimilation rate) of the control plants was nearly four-fold greater than that of plants grown with the highest 200 mM NaCl salt treatment (Table 1; Figure 4a). The stomatal conductance and transpiration rate appeared to have a similar response to the salt treatments, with a significant reduction with 200 mM NaCl compared to the controls (Table 1; Figure 4b,c). There was no significant difference in the gas exchange parameters (assimilation, conductance, and transpiration) between plants grown with the three lower levels of salt. No significant differences in dark-adapted chlorophyll fluorescence (F_v_/F_m_) or chlorophyll concentrations were detected between salt treatments either (Table 1), although we note that these measurements were taken on the second fully expanded leaf, which appeared to be unaffected by the salt treatment.

### 2.3. Minerals and Micronutrients

**Figure 5:**
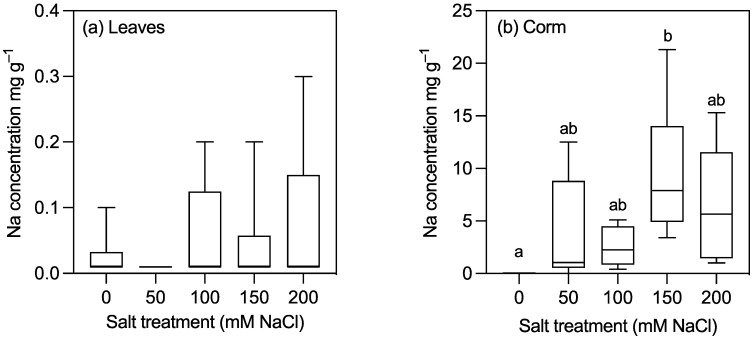
Sodium concentrations in (**a**) leaves and (**b**) corms of 30-week-old taro plants grown for 12 weeks with five different concentrations of sodium chloride. Columns are the means of six replicates ± 1 SE. Means with the same letter are not significantly different at *p* < 0.05.

The most striking observation was of the effect of the salt treatments on sodium concentrations. In corms, sodium concentrations increased with the salt treatments, with the sodium concentration ~70% higher in the 150 mM treatment group compared to the control (Table 2, Figure 5b). In the leaves, there were no significant differences in the sodium concentrations among the salt treatments, and 0.01 mg g^−1^ was the median sodium concentration in all the treatments (Table 2, Figure 5a). Six outliers were identified across the treatment groups, but these were not significant (Table 2). The remaining 26 plants all had a consistent sodium level of 0.01 mg g^−1^. The findings for sodium were consistent when nutrients were examined as both concentrations (mg g^−1^) and total contents per plant measures (Table 2, Supplementary Table S2).

**Figure 6:**
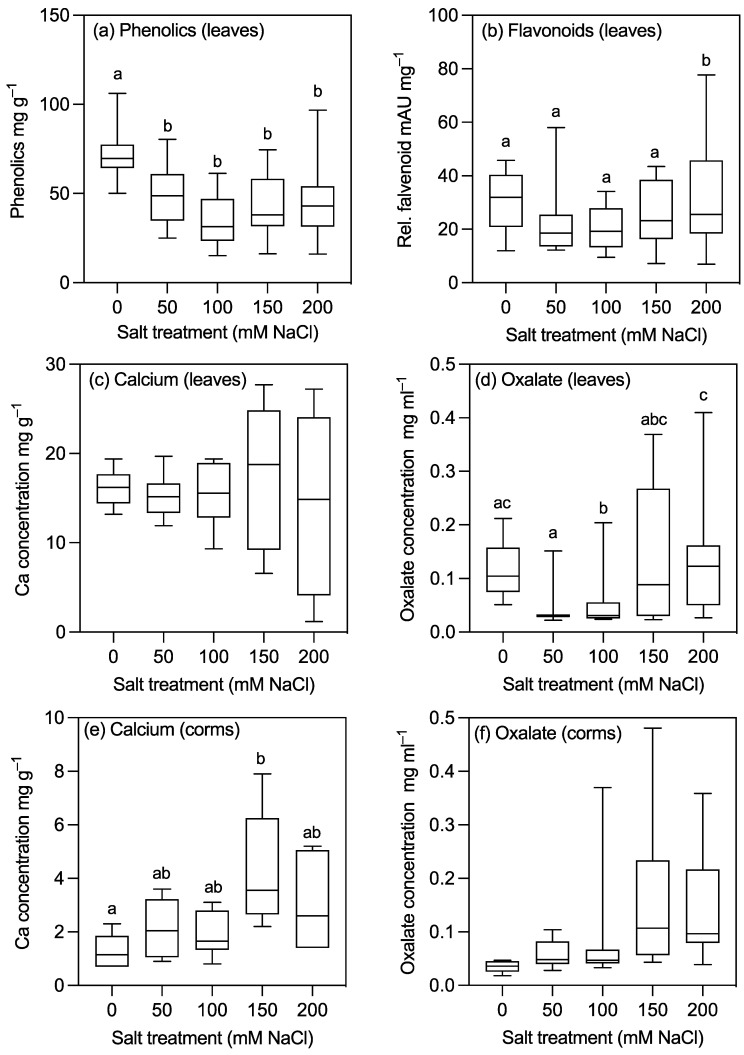
Concentrations of secondary metabolites and anti-nutritional factors in the leaves (**a**–**d**) and corms (**e**,**f**) of 30–week-old taro plants grown for 12 weeks at five different concentrations of sodium chloride. (**a**) Relative phenolic acid concentration (mAU mg g^−1^, *n* = 18); (**b**) relative flavonoid concentration (mAU mg^−1^, *n* = 18); (**c**) foliar calcium concentration (mg g^−1^, *n* = 6); (**d**) foliar oxalate concentration (mg ml^−1^, *n* = 18); (**e**) corm calcium concentration (mg g^−1^, *n* = 6); (**f**) corm oxalate concentration (mg ml^−1^, *n* = 18). Boxes for the same parameter with the same letter are not significantly different at *p* < 0.05.

There were significant differences in the concentrations of other leaf nutrients among the treatment groups, except for Zn, which was much more highly concentrated in plants growing in the 200 mM NaCl group compared to the controls (Table 2). By contrast, in the corms, the concentrations of most nutrients increased with the salt treatments (Table 2). For example, the concentrations of nitrogen and phosphorus were greater in plants in the 150 and 200 mM NaCl groups (Table 2). Importantly, for this study, in relation to calcium oxalate, the concentrations of Ca in the corms were approximately 30% higher in plants in the 150 mM NaCl treatment group than in the control plants (Figure 6).

In order to test whether the increases in concentrations of the corm and leaf nutrients in the plants with the higher salt treatments were the result of the reduced biomass of these plants, the total mass of each nutrient was calculated on a whole plant basis by multiplying the nutrient concentrations (mg g^−1^) by the corresponding corm and leaf mass. When the corm nutrients were examined as the total corm nutrients per plant, the eight nutrients that had increased in concentration were no longer significantly different between treatments (Supplementary Table S2). Only the corm carbon and sodium contents were significantly different among the treatments, with the carbon content decreasing with increasing salt concentrations, and total salt content increasing (Supplementary Table S2). Thus, the increases in nutrient concentrations per gram were the result of a smaller tissue mass with a similar overall amount of the various ions. When leaf nutrients were examined as total leaf nutrients per plant, all measured nutrients were actually lower in plants with the various salt treatments than in the control, aside from zinc and sodium (Supplementary Table S2).

### 2.4. Oxalate Determination

The concentrations of oxalate and calcium in leaves and corms were determined as a proxy for calcium oxalate (Figure 6; Table 2; Supplementary Tables S2 and S3). There were no significant differences in oxalate concentrations in the corms, whereas leaf oxalate concentrations were greater in the control plants than in plants in the 100 mM NaCl treatment group, and were lower in plants in the 100 and 50 mM NaCl treatment groups compared to those in the 200 mM NaCl treatment group (Figure 6; Supplementary Table S3). Higher degrees of variability were observed in the higher salinity treatment groups (i.e., 150 mM and 200 mM NaCl). For example, oxalate concentrations ranged between 0.051 and 0.212 mg ml^−1^ in the control treatment group, with a median value of 0.104 mg ml^−1^, compared to a range of 0.027–0.41 mg ml^−1^ and a median value of 0.028 mg ml^−1^ in the 200 mM treatment group. When leaf oxalates were examined as the total leaf oxalate content on a per plant basis, oxalate contents were lower in the salt treatment groups compared to the control (Supplementary Table S3). Similarly, leaf calcium contents were lower in the 150 and 200 mM NaCl plants compared to the control and in the 200 mM NaCl plants compared to the 50 mM NaCl plants (Supplementary Table S2).

### 2.5. Specialised Metabolites

Cyanogenic glycosides were detected in the leaf samples, but the concentrations were very low. The mean foliar cyanide concentrations of plants on a dry mass basis were 0.32 ± 0.10 μg HCN g^−1^, with values ranging from 0 to 1.46 μg g^−1^. There were no significant differences in the concentration of foliar cyanide or the total foliar cyanide content per plant (i.e., concentration × leaf mass) between salt treatments (Figure 6a; Supplementary Table S3).

Phenolic acid concentrations decreased with increasing salt concentrations and were lower in plants with 200 mM NaCl compared to those in the control and those with lower salt treatments (Figure 6c). By contrast, flavonoid concentrations were greater in plants with 200 mM NaCl than in those with 100 mM NaCl (Figure 6d). The relative effects of salinity on phenolics and flavonoids in leaves and corms were also significant when represented on a whole plant basis (Supplementary Table S3). The phenolic acid and flavonoid contents were greater in the control compared to the salt treatment groups, in the 50 mM NaCl groups compared to the 150 and 200 mM NaCl treatment groups, and in the 100 mM group compared to the 200 mM NaCl treatment group.

## 3. Discussion

We measured the effects of salt on the composition and growth of taro watered with different concentrations of salt. The key effects of salinity on taro were a reduction in biomass and photosynthetic rate. There were increases in the concentrations of several key corm nutrients due to reduced biomass, although the total content of mineral nutrients per plant organ did not increase.

### 3.1. Sodium Excluded from Upper Parts of Plant but Not Corms

The tissue sodium concentration increased in the corms with increasing salinity but not in the leaves (Table 2). This suggests that while salt may gain entry to the plant in the transpiration stream, it is excluded from the leaves. These observations are consistent with those of Hill et al. [13], who found that leaf sodium concentrations in taro grown hydroponically remained reasonably constant with increasing salt concentrations (0–80 mM NaCl), whereas the sodium concentration in the petioles and roots increased with increasing salt concentrations.

Sodium exclusion from upper plant parts has more generally been associated with salt tolerance in several plant species [3,18], including durum wheat and Indica rice cultivars, which can exclude sodium up to ~100 mM NaCl [25,26]. Low leaf sodium concentrations are often characteristic of non-halophytic species (i.e., those that are not adapted to growing in highly saline environments), with 200 mM considered the limit of growth for such species [16]. A modest degree of sodium exclusion has also been observed in the leaves of cassava [1]. However, the breakdown of this exclusion mechanism at relatively low concentrations (40 mM NaCl) suggests that the sodium exclusion mechanism present in taro is more effective [1]. Here, while taro was able to continue to grow and exclude sodium from transpiring leaves with 200 mM NaCl, an increase in senescent leaves, marginal leaf chlorosis, and scorching were observed in the highest salt treatment groups at harvest. This suggests that taro’s ability to exclude sodium from leaves may break down in the oldest leaves of the 150 and 200 mM NaCl plants after long-term exposure to saline conditions. The oldest leaves display the effects of salt toxicity first, probably because they have been transpiring for longer, and thus, have accumulated Na^+^ for longer [27,28].

### 3.2. Growth and Photosynthesis Are Reduced in Plants Grown at Moderate Salinity

Taro exhibited significant reductions in growth and biomass with increasing salinity, although the concentrations of salt at which the effects were manifested differed between the various parameters (between 50 mM and 200 mM). These results are broadly consistent with those from the in vitro studies on taro by Nyman and others who reported significant growth reduction and necrosis in taro cell cultures grown with 175 mM NaCl [24,29]. These results are also consistent with field observations made in Tuvalu of giant swamp taro (*Cyrtosperma merkusii*), a related edible aroid; plants grew well in locations where salt was ~100 mM NaCl and tolerated levels up to ~200 mM NaCl, but salt levels above 300 mM NaCl could be lethal [30].

The Fv/Fm decreased with increasing salinity, but the difference was not significant. The net carbon assimilation also decreased significantly with increasing salinity, with a significant decrease in all gas exchange parameters in plants grown with 200 mM NaCl. That the growth was impacted to a greater extent than the net carbon assimilation is not surprising; a small change in photosynthesis over time can result in highly significant reductions in biomass. It is also possible that the plants were expending more energy on other metabolic activities, such as ion exclusion at the level of the leaves (Table 2, Leaf). Reductions in growth and photosynthesis may be the result of physiological drought caused by osmotic stress rather than any direct toxic effects of sodium and chloride ions [14,31]. This conjecture is supported by the observed decrease in the rate of transpiration and stomatal conductance in the plants grown with higher levels of salt. Both salt exclusion and osmotic adjustment impose a high metabolic cost, which requires the diversion of assimilate from growth processes [27]. Nevertheless, while the photosynthetically active leaves stayed healthy, there was a higher rate of senescence of the older leaves in plants grown with high levels of salt, resulting in the loss of photosynthetic area. Thus, while tolerance mechanisms appear to allow for the continued survival of taro in saline conditions, the operation of these mechanisms may contribute to salinity-induced reductions in biomass, suggesting the existence of a trade-off between the yield and survival of taro [32].

Corm growth was more sensitive to salt than other plant parts, with reductions in biomass of approximately 30% observed in plants grown with the lowest concentration of 50 mM NaCl. This is not surprising, given the reduction in photosynthesis and the availability of carbohydrates for storage [32]. Other plants with underground storage organs, such as potato [33] and cassava [1], also exhibit significant yield reductions of 42–59%, at 70–80 mM NaCl. The effects of salinity on the biomass of the tuberous roots of cassava are particularly severe if the saline conditions coincide with the periods of tuber initiation and development [1]. The present study was conducted during the critical period when the storage organs were forming (4–7 months after planting), and this may explain the large reductions in corm yield observed [11]. Given that sodium concentrations were found to increase in corms with higher salt treatments, it is possible that the growth reductions observed in taro corms are the result of both osmotic and ionic effects, which culminate in the significant stunting of growth [28,31]. Our findings place taro among the salt-tolerant non-halophytes, plants that exhibit moderate salt tolerance while relying exclusively on salt exclusion [15,16]. The current study suggests that taro is significantly more tolerant to salinity than other regionally important tuberous crops, such as cassava and sweet potato, which both exhibited significant reductions in biomass at much lower concentrations of NaCl than shown here for taro [1,7,34].

### 3.3. Mineral Nutrient Uptake Decreased with Increasing Salinity

The concentrations of mineral nutrients recorded in taro corms in this study are broadly comparable to those recorded in previous studies [35,36]. The total amount of key mineral nutrients taken up overall in the corms (on a per mass basis) was lower in plants with the highest salt treatments (i.e., grams per total mass), even though the concentrations were higher. This can be attributed to the lower overall mass, that is, a concentration effect, as well as the lower transpiration rates in plants growing in the saline soil [37]. Foliar concentrations of macronutrients were also in the expected range for taro [38], except for Ca and K, which approached limiting levels for growth [39]. The high levels of Na^+^ and Cl^-^ ions in the soil may compete directly with the uptake of essential nutrients, such as Ca^2 +^, K^+^, and NO_3_^−^ [31,37]. This effect is thought to be more pronounced in taro because the mechanisms to exclude sodium from transpiring leaves may also restrict the movement of other essential nutrients through the plant [37].

### 3.4. Calcium Oxalate Crystal Formation and Remobilisation May Increase Survival at High Levels of NaCl

Concentrations of oxalate and calcium were measured as a proxy for calcium oxalate. Foliar oxalate and calcium concentrations decreased both on a per mass and a per organ basis with increasing salinity despite the decrease in plant biomass. The lower concentrations could be due to either a decrease in available Ca due to competition with sodium at the root, reduced transportation of Ca to the leaves as a result of reduced transpiration, or a reduction in oxalate formation. Evidence to support the latter hypothesis regarding Ca^2+^, salinity, and reduced CaOx accumulation comes from the study performed by Hunsch et al. [40], who found that a decrease in Ca^2+^ accumulation in the leaves of *Grewia tenax* (a salt-tolerant tropical tree) in response to high salinity resulted in the production of fewer CaOx crystals. In the same study, they included another tropical tree that does not accumulate CaOx crystals (*Tamarindua indica*), but in this case, they did not detect any difference in foliar Ca^2+^ levels. Thus, decreases in CaOx crystals are likely to be driven by a reduction in oxalate formation.

Alternatively, it is possible that any crystals in the leaves were remobilized. Remobilization would have the benefit of releasing oxalate back into the primary metabolism [41]. It is possible that the release of carbon dioxide from the breakdown of oxalate could make up for the reduction in gas exchange, allowing photosynthesis to continue for longer in stressed plants despite the partial closure of the stomata, both mitigating the impact on growth and consuming excess energy from the electron transport chain, and thus, limiting the accumulation of reactive oxygen species [42]. The finding that there was a reduction in the electron transport in the leaves of the tree that accumulated CaOx crystals (*G. tenax*) but not in the tree without this capacity supports this hypothesis [40]. If this mechanism operates in taro under salt stress, it could explain why, in our study, taro was able to maintain its growth rate with the 50 mM NaCl treatment and continue to photosynthesize up to 150 mM NaCl.

### 3.5. Secondary Metabolites in Plants Grown with Higher Concentrations of Salt

There was a significant reduction in phenolic acids and an increase in flavonoids in the taro leaves in response to increased salinity. This is in direct contrast to previous research on the response of secondary metabolites to stress, which have been observed to increase in concentrations in a wide range of crop plants, including rice, red pepper, and cassava [1,19,20,43]. The difference may be due, in part, to the length and magnitude of the stress imposed. Stress induces the upregulation of a variety of genes and protective systems to minimize plant damage, such as the salt exclusion mechanism observed in our study. However, these processes require a large energy expenditure [41] and may not be advantageous in the long term or with high levels of stress [41,42,44,45].

This study confirms that taro is cyanogenic, but the cyanide levels recorded are very low (1 μg g^−1^). Our results are broadly consistent with those previously recorded for taro by [46], who reported foliar cyanide levels of 0–3 mg HCN/100 g fresh weight. Other cyanogenic crops plants, such as cassava and sorghum, have much higher foliar cyanide concentrations, ranging from 0.2 to 20 mg g^−1^ [47,48]. There was no significant difference found in the HCN concentrations of taro plants among the salt treatments. This is in contrast to prior studies on other cyanogenic species, such as cassava and white clover, which reported significant increases in foliar cyanide concentrations at salt concentrations up to 40 mM NaCl in both species, and a subsequent decrease at 80 mM NaCl in cassava [1,49].

### 3.6. Conclusions and Implications for Food Security

The increasing demand for food due to population growth, together with declining yields, poses a significant threat to food security in the Pacific region, particularly given the salt-sensitive status of other regionally important crops, such as cassava and sweet potato. Taro is moderately tolerant to salt, with little impact on size and morphology when grown with up to 100 mM NaCl, and can survive with up to 200 mM NaCl. Taro will continue to be nutritious, however, significant reductions in growth and biomass are of concern to the future of taro production in regions subject to rising sea levels and ground water salinization. Breeding for more salt-tolerant varieties may be necessary for taro to continue its role as a staple in the Pacific under future saline conditions.

## 4. Materials and Methods

### 4.1. Plant Material, Glasshouse Conditions, and Treatment Groups

A glasshouse experiment was conducted at the School of Biological Sciences, Plants Sciences Complex, Monash University, Clayton. Taro plantlets (*Colocasia esculenta* (L.) Schott cv. Samoan Pink), approximately 20 cm in size and sourced from El Arish Tropical Exotics (Queensland, Australia), were grown in 20 L pots containing commercial Debco potting mix and Osmocote^®^ controlled-release fertilizer (Scotts, Marysville, Ohio, USA; containing NPK 19.4:1.6:5 and micronutrients) under natural light. The plants were watered with a complete nutrient solution (Searles^®^, Winya, Queensland, Australia; 10 mL l^−1^ H_2_O) at 0, 30, and 60 days after transplanting (DAT). The temperature in the greenhouse was maintained at 26 and 20 °C during the day and night, respectively, with a range of 22 °C to 30 °C and humidity between 55 and 65%, consistent with the optimal conditions for taro reported in the literature [11]. The plants were rotated once a week around the greenhouse to minimize vagaries in the microenvironment.

After 18 weeks of growth (12 December 2016 to 20 March 2017), the taro plants were classified into three groups based on height (small: approximately 20–35 cm; medium: approximately 35–45 cm; large: approximately 45–60 cm), and 18 plants within each group were randomly allocated to one of 5 salt treatments (0, 50, 100, 150, or 200 mM NaCl; *n* = 18), such that each treatment group had an equal number of plants from the small, medium, and large groups. Seawater has a concentration of approximately 500 mM NaCl. The salt concentrations were determined based on the studies of Nyman and Arditti [24] and Hill, Abaidoo, and Miyasaka [13], which indicate a maximum survivable concentration of between 80 mM and 175 mM. In order to avoid a shock response, the salt concentrations were increased gradually over two weeks, starting at 25 mM and increasing by 25 mM every 3 days until the final concentrations were reached, following the methods of Gleadow, Pegg, and Blomstedt [1]. The final concentrations were maintained for 10 weeks, and the plants were watered with 500 mL of the appropriate salt solution three times a week. The pots were flushed weekly to prevent salt build-up in the pots. The salt concentrations of leachate from a subset of samples (*n* = 3) were measured halfway through the treatment period (6 weeks) using a portable refractometer to confirm that the allocated salt concentrations were not exceeded (Supplementary Table S4).

### 4.2. Phenology, Harvesting, and Sampling Protocol

Plant height, leaf number, stage of leaf development, and width and length of the third fully expanded leaf blade were recorded weekly from the first application of the salt treatment for the duration of the experiment (12 weeks). The leaves were classified as peeping, rolled, expanded, or senescent following Lu et al. [50], as modified by Crimp et al. [51] (Supplementary Table S5).

The initial plant biomass was measured at the start of the study by harvesting a group of plants 18 weeks after transplanting (*n* = 15, equally distributed between height groups). The plants were grown under treatment conditions for 12 weeks before being destructively harvested when they were 7 months old. This time period was chosen because, at this age, plants typically have a well-developed and mature corm, but side shoot development is limited. The plants were separated into the leaves, petioles, roots, and corm. The leaves were further separated into the five classes described above (Supplementary Table S5). The leaf area was measured for expanded and expanding leaves using an LI-3000 Portable Leaf Area Meter (Li-Cor, Lincoln, NE, USA). The fresh weight was recorded for all plant parts (petioles, roots, corms) and leaves from each class. The second fully expanded leaf from each plant was freeze-dried for secondary metabolite and chlorophyll analyses. The remaining plant parts were oven-dried at 60 °C for 7 days and re-weighed for dry weight determination. Where present, side shoots (‘suckers’) were separated from the main stem, their plant parts sorted into roots, leaves, and petioles, as above, and the dry mass measured separately from the main plant. Growth indices were calculated, including the relative growth rate [51], the net assimilation rate (NAR), and the root/shoot ratio, following the methods of [1] (Supplementary Table S6).

### 4.3. Photosynthetic Parameters

The photosynthetic parameters were measured on four representative plants from each treatment group in the week prior to the final harvest. The photosynthetic rate, transpiration, conductance, and internal CO_2_ concentration were measured using a Li-Cor 6400 portable photosynthesis machine (Li-Cor, Lincoln, NE, USA). Measurements on the second fully expanded leaf from each plant (*n* = 5) were made at 800 μmol quanta m^−2^ s^−1^ PAR, 400 ppm CO_2_, 25 °C, and approximately 50% relative humidity. Dark-adapted chlorophyll fluorescence, F_v_/F_m_, was measured on the second fully expanded leaf (*n* = 4) using a Pulse-Amplitude Modulated PAM-210 Chlorophyll Fluorometer (Walz, Effeltrich, Germany). The F_v_/F_m_ ratio is an indicator of the degree of potential photosynthetic ability and nutrient stress, where 0.8–0.9 is indicative of a healthy, unstressed plant [52].

### 4.4. Chemical Analysis of Primary and Secondary Metabolites

The chlorophyll concentrations were measured using the method of Gleadow et al. [53], as modified by Burns et al. [54]. Briefly, ground freeze-dried leaf tissue (0.020 g) was extracted twice in 80% acetone and the supernatants from the two extractions were pooled. The absorbance of the supernatant was measured at 450, 647, 665, and 750 nm using a spectrophotometer (Varian Cary© 50 Bio UV-Visible), and the concentrations of chlorophyll and carotenoids were calculated using the equations of Jaspars [55].

Micro- and macronutrient analysis of finely ground freeze-dried tissue (0.2 g) was conducted on a subset of leaf and corm samples (Table 2; *n* = 6 per plant part) using inductively coupled plasma mass spectrometry (ICPMS) (Environmental Analysis Laboratories, Southern Cross University, NSW).

Phenolics and oxalates were measured on finely ground freeze-dried corm and leaf tissue for each individual (*n* = 18) using reverse-phase high-performance liquid chromatography (HPLC) following the methods of Uesugi and Kessler [56]. For extraction, 1 mL of Milli-Q water (Milipore^®^, North Ryde, New South Wales, Australia) was added to 0.02 g of tissue. The samples were heated at 80 °C for 15 min, centrifuged for 30 min, and filtered through a 0.45 μm size pore membrane. Chromatographic analysis was carried out using an Agilent Technologies HPLC 1260 Infinity (Agilent Technologies, Waldbronn, Germany) using a reverse-phase Poroshell 120 column (particle size of 4.6 × 50 mM, 2.7 μm). Two solvents were used—0.25% H_3_PO_3_ in MQ H_2_0 (A) and acetonitrile (B), starting with 0% B and using a gradient to obtain 12% B at 2 min, 18% B at 3 min, 58% B at 10 min, and 0% B at 20 min. The flow rate was 0.5 mL/min, and the injection volume was 2 μL. Phenolic acids and flavonoids were identified to compound classes using UV spectra, and oxalates were identified based on the retention time and the UV spectra of the standard (Sigma© analytical standard, CAS Number: 144-62-7). Compounds were quantified at 210 nm for oxalates, 320 nm for phenolic acids, and 360 nm for flavonoids. The relative concentration of each compound was expressed as the peak intensity relative to the tissue mass of each sample, and the totals of each compound class were calculated. The concentrations of oxalic acid were quantified using a standard curve of 0.1–0.02 mg ml^−1^ oxalic acid.

A subset of plants in the experiment (*n* = 5) were tested for the presence of cyanogenic glycosides using the evolved cyanide and capture method [48,57]. Triglochinin, the cyanogenic glucoside in taro [22], is an aliphatic cyanogenic glucoside and is unlikely to be catalysed by commercially available β-glucosidases that are particularly effective against aromatic amino acids [58]. To ensure the hydrolyzation of all cyanogenic glycosides present in taro tissues, β-glucosidase emulsion (β-D-glucoside glucohydrolase; EC 3.2.1.21) from almond (*Prunus amygdalis* (L.) Benth. Hook.) and a solution of latex from cassava (*Manihot esculenta* Cranz), previously shown to be effective against aliphatic amino acids [1,59], were used. The β-glucosidase (200 μL) and latex solution (200 μL suspended in 0.1 M of phosphate buffer with a pH of 6.0) were combined with freeze-dried tissue (0.1 g) in sealed vials. Separate tubes containing 200 μL of 1 M NaOH were inserted into the vials to act as a trap for the evolved hydrogen cyanide (HCN). The vials were frozen and thawed at room temperature to disrupt the cells and allow the cell contents to mix, and then incubated for 16 h at 37 °C [60]. Hydrogen cyanide in the NaOH was assayed colorimetrically in 96-well titre plates using a FLUOstar Galaxy plate reader (BMG, Australia) with NaCN in 0.1 M of NaOH as the standard. One gram of HCN is equivalent to 13.3 g of triglochinin.

### 4.5. Statistical Analysis

Data analysis was performed using R version 4.1.1 and RStudio version 1.4.1717 [61]. The weekly phenology data were analysed using linear mixed-effect models, with ‘salt treatment’, ‘week of treatment’, and ‘initial height block’ as the fixed effects and ‘individual’ as a random effect to account for repeated measures. The measurements relating to plant suckers were analysed using Kruskal–Wallis tests, as the data were non-parametric. The remaining data were analysed using ANOVAs, in which ‘days of growth’ was used as a covariate to control for different harvest dates. The means were compared using post-hoc Tukey’s honest significant difference tests, by which statistical significance was detected among the groups. Where a significant interaction was found between predictors in the LME models, the data were subset by ‘week of treatment’. Square root and log transformations were applied where required in order to satisfy the assumptions of normality and equal variances. A rank normal transformation was applied to three variables (the root/shoot ratio, and the foliar carbon and calcium concentrations) where deviation from normality was more severe. All significance testing was at the 0.05 level.

## Figures and Tables

**Table 1:** Summary of the mean values and ANOVA results for growth (*n* = 18) and photosynthetic (*n* = 4) variables in taro plants grown with five salt concentrations (0, 50, 100, 150, and 200 mM NaCl) for 12 weeks.

Growth Parameter	0 mM	50 mM	100 mM	150 mM	200 mM	*df*	*F*	*p*
Total biomass (g)	32.05 ± 3.60 ^a^	24.04 ± 2.35 ^b^	17.44 ± 1.50 ^bc^	10.93 ± 0.94 ^c^	14.10 ± 2.10 ^bc^	4	14.54	0.001 *
Leaf mass (g)	3.26 ± 0.52 ^a^	2.70 ± 0.35 ^ab^	1.83 ± 0.17 ^b^	0.91 ± 0.15 ^c^	0.35 ± 0.05 ^c^	4	21.03	0.001 *
Petiole mass (g)	5.72 ± 1.14 ^a^	4.84 ± 0.66 ^ab^	2.75 ± 0.31 ^bc^	1.28 ± 0.19 ^cd^	0.60 ± 0.12 ^d^	4	19.32	0.001 *
Root mass (g)	2.77 ± 0.18 ^a^	2.34 ± 0.38 ^ab^	1.66 ± 0.14 ^bc^	1.20 ± 0.19 ^c^	1.05 ± 0.11 ^c^	4	12.39	0.001 *
Corm mass (g)	20.31 ± 2.41 ^a^	14.19 ± 1.56 ^b^	11.20 ± 1.32 ^b^	7.55 ± 0.74 ^b^	12.11 ± 1.98 ^b^	4	8.21	0.001 *
Above-ground mass (g)	8.97 ± 1.64 ^a^	7.53 ± 1.00 ^ab^	4.58 ± 0.47 ^bc^	2.19 ± 0.33 ^c^	0.95 ± 0.16 ^d^	4	20.57	0.001 *
Below-ground mass (g)	23.08 ± 2.46 ^a^	16.51 ± 1.80 ^b^	12.86 ± 1.38 ^b^	8.74 ± 0.79 ^b^	13.15 ± 2.01 ^b^	4	9.76	0.001 *
Plant height (cm)	69.06 ± 3.14 ^a^	59.58 ± 5.58 ^b^	57.58 ± 1.97 ^ab^	40.32 ± 3.16 ^c^	28.50 ± 3.06 ^c^	4	21.40	0.001 *
Leaf number	3.67 ± 0.16 ^a^	3.00 ± 0.29 ^ab^	3.28 ± 0.20 ^ab^	2.71 ± 0.27 ^bc^	1.72 ± 0.14 ^d^	4	10.41	0.001 *
No. senescent	3.22 ± 0.15 ^a^	2.72 ± 0.30 ^a^	2.83 ± 0.20 ^a^	1.18 ± 0.21 ^b^	0.94 ± 0.17 ^a^	4	5.66	0.001 *
No. fully expanded	0.11 ± 0.08 ^a^	0.22 ± 0.10 ^a^	0.11 ± 0.08 ^a^	0.82 ± 0.15 ^b^	0.33 ± 0.11 ^b^	4	23.16	0.001 *
Leaf area (cm^2^)	1007.07 ± 144.03 ^a^	821.67 ± 108.30 ^ab^	511.18 ± 53.77 ^b^	143.73 ± 22.83 ^c^	79.86 ± 15.90 ^c^	4	31.06	0.001 *
Root/shoot ratio	3.38 ± 0.57 ^a^	5.19 ± 2.30 ^a^	3.37 ± 0.51 ^a^	5.70 ± 0.96 ^a^	20.71 ± 3.55 ^b^	4	11.28	0.001 *
RGR (g g^−1^ day^−1^)	0.003 ± 0.00 ^a^	0.001 ± 0.00 ^ab^	0.000 ± 0.00 ^bc^	−0.002 ± 0.00 ^c^	−0.002 ± 0.00 ^c^	4	13.40	0.001 *
LAR (cm^2^ g^−1^)	32.31 ± 2.57	36.47 ± 3.70	31.00 ± 3.06	14.93 ± 3.32	6.56 ± 1.75	4	1.60	0.181
NAR (g cm^2^ day^−1^)	0.0002 ± 0.00 ^a^	0.0002 ± 0.00 ^ab^	0.0002 ± 0.00 ^a^	0.0003 ± 0.00 ^ab^	0.0004 ± 0.00 ^b^	4	4.791	0.002 *
A (µmol CO_2_ m^−2^ s^−1^)	9.36 ± 1.06 ^a^	7.25 ± 0.94 ^ab^	4.59 ± 1.41 ^ab^	5.84 ± 1.12 ^ab^	2.60 ± 1.00 ^b^	4	4.99	0.012 *
g_s_ (mmol H_2_O m^−2^ s^−1^)	0.10 ± 0.03 ^a^	0.06 ± 0.01 ^ab^	0.04 ± 0.01 ^ab^	0.05 ± 0.01 ^ab^	0.02 ± 0.01 ^b^	4	4.99	0.028 *
T (mmol H_2_O m^−2^ s^−1^)	2.29 ± 0.72 ^a^	1.28 ± 0.35 ^ab^	0.66 ± 0.13 ^ab^	1.00 ± 0.14 ^ab^	0.38 ± 0.17 ^b^	4	3.84	0.012 *
Fv/Fm	0.88 ± 0.01	0.87 ± 0.00	0.84 ± 0.05	0.79 ± 0.04	0.67 ± 0.11	4	1.24	0.367
Total Chlorophyll (mg g^−1^)	12.33 ± 1.06	9.46 ± 1.33	10.08 ± 0.44	10.41 ± 0.87	10.30 ± 0.98	4	1.95	0.210

Table footer: * RGR = relative growth rate, LAR = leaf area ratio, NAR = net assimilation rate, A = carbon assimilation rate, *g_s_* = stomatal conductance, T = transpiration. Values are means ± 1 SE. Comparisons that are significantly different (*p* < 0.05) are indicated with an asterisk (*). Means with the same letter are not significantly different at *p* < 0.05.

**Table 2:** Macro and micronutrients of oven-dried leaves and corms of taro plants supplied with five different concentrations of salt (0, 50, 100, 150, and 200 mM NaCl) for 12 weeks. Values are the means of six replicates ± 1 SE in mg g^−1^. Comparisons that are significantly different (*p* < 0.05) are indicated with an asterisk (*). Means with the same letter are not significantly different at *p* < 0.05.

Leaf								
	0 mM	50 mM	100 mM	150 mM	200 mM	*df*	*F*	*p*
Nitrogen	37.00 ± 1.64	43.35 ± 2.59	43.03 ± 2.56	44.45 ± 3.53	43.42 ± 3.63	4	1.13	0.367
Phosphorus	4.07 ± 0.41	4.82 ± 0.70	4.55 ± 0.33	5.02 ± 0.44	4.23 ± 0.50	4	0.61	0.658
Potassium	35.40 ± 2.24	33.10 ± 2.63	32.28 ± 2.10	35.82 ± 2.63	33.67 ± 3.78	4	0.24	0.915
Sulphur	2.10 ± 0.08	2.47 ± 0.15	2.48 ± 0.12	2.52 ± 0.23	2.42 ± 0.22	4	1.09	0.385
Carbon	431.17 ± 3.13	425.83 ± 1.92	430.50 ± 4.25	424.83 ± 10.35	419.67 ± 10.49	4	0.12	0.975
Calcium	16.15 ± 0.89	15.22 ± 1.06	15.43 ± 1.53	17.63 ± 3.38	14.37 ± 4.08	4	0.29	0.880
Magnesium	4.28 ± 0.23	3.73 ± 0.28	3.52 ± 0.20	4.03 ± 0.41	3.93 ± 0.75	4	0.46	0.768
Sodium	0.03 ± 0.02	0.01 ± 0.00	0.06 ± 0.03	0.04 ± 0.03	0.07 ± 0.05	4	0.78	0.549
Copper	0.01 ± 0.00	0.01 ± 0.00	0.01 ± 0.00	0.01 ± 0.00	0.01 ± 0.00	4	2.79	0.052
Zinc	0.03 ± 0.01 ^a^	0.04 ± 0.01 ^ab^	0.07 ± 0.01 ^ab^	0.07 ± 0.01 ^ab^	0.08 ± 0.01 ^b^	4	4.06	0.013 *
Manganese	0.12 ± 0.01	0.12 ± 0.01	0.11 ± 0.01	0.12 ± 0.02	0.10 ± 0.02	4	0.20	0.933
Iron	0.16 ± 0.03	0.06 ± 0.01	0.06 ± 0.02	0.06 ± 0.02	0.04 ± 0.01	4	2.01	0.128
**Corm**								
	**0 mM**	**50 mM**	**100 mM**	**150 mM**	**200 mM**	***df***	***F***	***p***
Nitrogen	3.38 ± 0.47 ^a^	6.05 ± 1.10 ^ab^	5.38 ± 2.03 ^ac^	20.75 ± 4.06 ^b^	20.05 ± 7.40 ^bc^	4	5.47	0.003 *
Phosphorus	1.87 ± 0.15 ^a^	1.78 ± 0.24 ^a^	2.03 ± 0.48 ^a^	6.82 ± 1.00 ^b^	5.50 ± 1.47 ^b^	4	9.06	0.001 *
Potassium	10.22 ± 1.18	10.62 ± 1.20	12.15 ± 2.01	8.95 ± 1.04	9.13 ± 0.99	4	0.92	0.472
Sulphur	0.37 ± 0.06	0.53 ± 0.10	0.47 ± 0.13	1.55 ± 0.21	1.57 ± 0.56	4	1.06	0.399
Carbon	437.50 ± 14.92	417.67 ± 21.87	424.17 ± 2.98	414.67 ± 2.93	416.33 ± 4.15	4	0.58	0.684
Calcium	1.28 ± 0.26 ^a^	2.13 ± 0.48 ^ab^	1.90 ± 0.35 ^ab^	4.28 ± 0.88 ^b^	3.03 ± 0.74 ^ab^	4	3.76	0.019 *
Magnesium	1.13 ± 0.11 ^a^	1.07 ± 0.17 ^a^	0.93 ± 0.14 ^a^	2.50 ± 0.24 ^b^	1.82 ± 0.43 ^ab^	4	6.68	0.001 *
Sodium	0.06 ± 0.02 ^a^	3.82 ± 2.08 ^ab^	2.55 ± 0.79 ^ab^	9.58 ± 2.62 ^b^	6.58 ± 2.34 ^ab^	4	4.05	0.013 *
Copper	0.003 ± 0.00 ^a^	0.005 ± 0.00 ^a^	0.003 ± 0.00 ^a^	0.010 ± 0.00 ^b^	0.006 ± 0.00 ^ab^	4	5.70	0.003 *
Zinc	0.04 ± 0.01	0.0676 ± 0.0143	0.06 ± 0.010	0.18 ± 0.07	0.07 ± 0.02	4	2.38	0.080
Manganese	0.01 ± 0.00	0.02 ± 0.01	0.03 ± 0.01	0.03 ± 0.01	0.02 ± 0.01	4	2.25	0.097
Iron	0.03 ± 0.01 ^a^	0.08 ± 0.01 ^ab^	0.11 ± 0.02 ^ab^	0.18 ± 0.04 ^b^	0.11 ± 0.02 ^b^	4	5.72	0.004*

## Data Availability

Data is contained within the article or supplementary material. Raw data is available from the corresponding author on request.

## Supplementary Materials

The following are available online at https://www.mdpi.com/article/10.3390/plants10112319/s1, Table S1. Statistical analysis for sucker growth variables; Table S2. Macro- and micronutrients of leaves and corms; Table S3. ANOVA results for secondary metabolites and anti-nutritional factors; Table S4. Concentration of salt in leachate from weekly freshwater flush of taro pots; Table S5. Classification of taro leaves based on their developmental stage; Table S6. Formulas for calculating growth indices.
