# Supplementary material for: Effects of Salinity on the Growth and Nutrition of Taro (Colocasia esculenta): Implications for Food Security
Source: Plants (Basel). 2021 Oct 28;10(11):2319. doi: 10.3390/plants10112319 (PMC8621212; doi:10.3390/plants10112319)
Supplement: Supplementary file 1 [file plants-10-02319-s001.zip › plants-1384643-supplementary.pdf]

## Supplementary Information

# Effects of Salinity on the Growth and Nutrition of Taro (*Colocasia esculenta*): Implications for Food Security

Georgia R. Lloyd, Akane Uesugi and Roslyn M. Gleadow

### Six tables are included as supplementary information

Supplementary Table S1. Statistical analysis for sucker growth variables

Supplementary Table S2. Macro and micronutrients content of leaves and corms of taro on a per organ basis

Supplementary Table S3. Statistical analysis for secondary metabolites and anti-nutritional factors

Supplementary Table S4. Concentration of salt in leachate from taro pots

Supplementary Table S5. Classification of taro leaves

Supplementary Table S6. Calculations for determination of growth indices

**Supplementary Table S1.** Summary of Kruskal-Wallis results for sucker growth variables (n=18) in taro plants grown at five salt concentrations (0, 50, 100, 150, 200 mM NaCl) for 12 weeks. Where % Sucker = % of biomass in sucker, sucker corm: main corm = ratio of sucker corm to main plant corm. Significance level $p<0.05$.

| | **Chi-squared** | ***df*** | ***p* value** |
|---|---|---|---|
| Sucker biomass | 5.87 | 4 | 0.21 |
| No. of Suckers | 3.83 | 4 | 0.43 |
| % Sucker | 3.59 | 4 | 0.46 |
| Sucker corm: main corm | 3.96 | 4 | 0.41 |

**Supplementary Table S2.** Macro and micronutrients of oven dried leaves **(a)** and corms **(b)** of taro plants supplied with five different concentrations of salt (0, 50, 100, 150, 200 mM NaCl) for 12-weeks. Values are the mean of six replicates ± 1SE in mg and represent nutrient content per total leaf and total corm mass per plant. Comparisons that are significantly different are indicated with an asterisk (*). Means with the same letter are not significantly different at *p*<0.05.

**Leaf**

| | **0mM** | **50mM** | **100mM** | **150mM** | **200mM** | ***df*** | ***f* value** | ***p* value** |
|---|---|---|---|---|---|---|---|---|
| Nitrogen | 160.94±51.84 **a** | 134.05±24.79 **a** | 70.33±11.50 **ab** | 35.53±9.33 **b** | 24.91±4.67 **b** | 4 | 7.87 | <0.001 * |
| Phosphorus | 15.63±4.28 **a** | 14.84±3.01 **a** | 7.43±1.32 **ab** | 4.40±0.94 **b** | 2.47±0.51 **b** | 4 | 9.67 | <0.001 * |
| Potassium | 141.96±41.22 **a** | 99.29±13.42 **a** | 54.50±11.56 **ab** | 26.81±5.88 **b** | 19.72±3.64 **b** | 4 | 10.10 | <0.001 * |
| Sulphur | 9.29±3.13 **a** | 7.73±1.58 **a** | 4.09±0.69 **ab** | 2.04±0.56 **b** | 1.40±0.27 **b** | 4 | 8.82 | <0.001 * |
| Carbon | 1849.14±577.20 **a** | 1284.12±171.09 **a** | 731.05±131.94 **ab** | 333.91±87.72 **b** | 237.34±33.27 **b** | 4 | 11.88 | <0.001 * |
| Calcium | 68.01±20.02 **a** | 47.30±9.91 **ac** | 27.72±5.71 **ab** | 14.21±5.61 **bc** | 8.56±2.69 **b** | 4 | 7.23 | <0.001 * |
| Magnesium | 19.05±6.24 **a** | 11.70±2.49 **ac** | 6.17±1.19 **ab** | 3.17±1.02 **bc** | 2.31±0.52 **b** | 4 | 7.90 | <0.001 * |
| Sodium | 0.06±0.02 | 0.03±0.00 | 0.11±0.09 | 0.02±0.01 | 0.03±0.02 | 4 | 1.66 | 0.198 |
| Copper | 0.03±0.01 **ab** | 0.03±0.01 **a** | 0.02±0.00 **ab** | 0.01±0.00 **b** | 0.01±0.00 **b** | 4 | 4.57 | 0.008 * |
| Zinc | 0.10±0.03 | 0.12±0.03 | 0.10±0.03 | 0.05±0.01 | 0.05±0.01 | 4 | 2.10 | 0.115 |
| Manganese | 0.44±0.13 **a** | 0.33±0.06 **a** | 0.19±0.04 **ab** | 0.09±0.03 **b** | 0.06±0.01 **b** | 4 | 8.29 | <0.001 * |
| Iron | 0.50±0.25 **a** | 0.18±0.05 **ab** | 0.11±0.04 **ab** | 0.06±0.02 **b** | 0.02±0.01 **b** | 4 | 7.60 | <0.001 * |

**Corms**

| | **0mM** | **50mM** | **100mM** | **150mM** | **200mM** | ***df*** | ***f* value** | ***p* value** |
|---|---|---|---|---|---|---|---|---|
| Nitrogen | 83.73±20.46 | 98.91±23.27 | 68.07±38.03 | 161.60±50.88 | 192.83±95.05 | 4 | 2.19 | 0.104 |
| Phosphorus | 47.53±10.11 | 31.83±6.69 | 25.78±10.19 | 51.13±10.78 | 58.46±21.84 | 4 | 1.38 | 0.274 |
| Potassium | 253.72±54.57 | 186.26±45.33 | 130.55±25.64 | 70.31±15.68 | 131.30±45.43 | 4 | 2.68 | 0.058 |
| Sulphur | 8.55±1.75 | 9.31±2.92 | 5.79±2.59 | 12.29±3.39 | 14.80±6.85 | 4 | 1.52 | 0.233 |
| Carbon | 11389.81±2620.52**a** | 7157.40±1178.88**ab** | 4709.74±855.35 **ab** | 3289.02±651.01 **b** | 5528.68±1539.79**ab** | 4 | 4.29 | 0.010 * |
| Calcium | 30.95±9.44 | 30.89±5.32 | 18.95±3.19 | 29.54±3.87 | 33.13±11.77 | 4 | 0.69 | 0.605 |
| Magnesium | 27.45±5.69 | 17.73±3.55 | 10.87±3.37 | 19.13±4.02 | 19.02±6.12 | 4 | 1.73 | 0.180 |
| Sodium | 1.45±0.73 **a** | 37.22±14.14 **ab** | 27.49±12.25 **ab** | 66.90±15.70 **b** | 53.71±20.13 **ab** | 4 | 7.42 | <0.001 * |
| Copper | 0.06±0.02 | 0.07±0.02 | 0.04±0.01 | 0.08±0.02 | 0.06±0.02 | 4 | 1.13 | 0.369 |
| Zinc | 0.92±0.30 | 1.05±0.22 | 0.65±0.17 | 1.26±0.40 | 0.72±0.29 | 4 | 0.23 | 0.583 |
| Manganese | 0.15±0.05 **a** | 0.25±0.08 **ab** | 0.24±0.05 **ab** | 0.20±0.04 **b** | 0.23±0.14 **ab** | 4 | 0.77 | 0.558 |
| Iron | 0.99±0.49 | 1.22±0.15 | 1.10±0.22 | 1.32±0.25 | 1.19±0.41 | 4 | 0.15 | 0.961 |

**Supplementary Table S3.** Summary of ANOVA results, for secondary metabolites and anti-nutritional factors in taro plants grown at five salt concentrations (0,50, 100, 150, 200 mM NaCl) for 12 weeks, represented as concentration and total content (content per total leaf and total corm mass per plant). Significance $p<0.05$.

| | *df* | *f* value | *p* value |
|---|---|---|---|
| **Concentration** | | | |
| Leaf Oxalate (mg/ml) | 4 | 8.10 | <0.001* |
| Corm Oxalate (mg/ml) | 4 | 1.86 | 0.126 |
| Leaf Phenolic acids (mAU/mg) | 4 | 13.44 | <0.001* |
| Leaf Flavonoids (mAU/mg) | 4 | 2.57 | 0.044 * |
| Leaf Cyanide (µg/g) | 4 | 1.36 | 0.282 |
| | | | |
| **Total content** | | | |
| Leaf Oxalate (mg) | 4 | 18.24 | <0.001* |
| Corm Oxalate (mg) | 4 | 1.20 | 0.320 |
| Leaf Phenolic acids (mAU) | 4 | 28.13 | <0001* |
| Leaf Flavonoids (mAU) | 4 | 17.06 | <0.001* |
| Leaf Cyanide (µg) | 4 | 1.61 | 0.211 |

**Supplementary Table S4.** Concentration of salt in leachate from weekly freshwater flush of taro pots (n=3) measured using refractometer. Results confirm that the soil salt concentration does not exceed target level. Concentration (mM NaCl) was calculated based on average concentration of seawater (Seawater = 3.5 % or 500mM NaCl).

| **Treatment (mM)** | **Replicate** | **% NaCl** | **~ mM NaCl** |
|---|---|---|---|
| 0 | 1 | 0 | 0 |
| 0 | 2 | 0 | 0 |
| 0 | 3 | 0 | 0 |
| 50 | 1 | 0 | 0 |
| 50 | 2 | 0 | 0 |
| 50 | 3 | 0.25 | 36 |
| 100 | 1 | 0.5 | 71 |
| 100 | 2 | 0.7 | 100 |
| 100 | 3 | 0 | 0 |
| 150 | 1 | 1 | 143 |
| 150 | 2 | 1 | 143 |
| 150 | 3 | 0.5 | 71 |
| 200 | 1 | 1 | 143 |
| 200 | 2 | 1.25 | 179 |
| 200 | 3 | 0.75 | 107 |

**Supplementary Table S5.** Classification of taro leaves**.** Leaves of taro were assigned to one of five classes based on their developmental stage (Crimp *et al.*, 2017).

| **Leaf type** | **Characteristics** |
|---|---|
| Peeping | Leaf emerging from sheath, tightly rolled |
| Rolled | Leaf blade folded in on itself |
| Expanding | Young, not fully unfurled |
| Expanded | Mature, fully developed leaf |
| Senescent | Yellow or brown in colour over >50% of leaf surface |

**Supplementary Table S6.** Calculations for determination of growth indices, where W=total plant mass, A=total leaf area and t=time, with 1 representing measurements taken at the beginning of the experiment and 2 those taken at the end (Gleadow and Rowan, 1982).

| **Growth index** | **Calculation** |
|---|---|
| Root/shoot Ratio | $\frac{\text{Total above– ground biomass}}{\text{Total below– ground biomass}}$ |
| LAR | $\frac{A2}{W2}$ |
| RGR | $\frac{\log W2 - \log W1}{\Delta t}$ |
| NAR | $\frac{W2 - W1}{\Delta t} \times \frac{\log A2 - \log A1}{A2 - A1}$ |
